# A Systematic Review of Gut Microbiota Diversity: A Key Player in the Management and Prevention of Diabetes Mellitus

**DOI:** 10.7759/cureus.69687

**Published:** 2024-09-18

**Authors:** Hema Manvi Koneru, Hooria Sarwar, Venkata Varshitha Bandi, Mohit Sinha, Pakeeza Tarar, Rafik Bishara, Iana Malasevskaia

**Affiliations:** 1 Internal Medicine, California Institute of Behavioral Neurosciences and Psychology, Fairfield, USA; 2 Psychiatry, California Institute of Behavioral Neurosciences and Psychology, Fairfield, USA; 3 Medical Research, California Institute of Behavioral Neurosciences and Psychology, Fairfield, USA; 4 Obstetrics and Gynecology, Private Clinic 'Yana Alexandr', Sana'a, YEM; 5 Research and Development, California Institute of Behavioral Neurosciences and Psychology, Fairfield, USA

**Keywords:** diabetes mellitus, dietary interventions, fecal microbiota transplantation, glycemic control, gut microbiota, metabolic disorders, microbiome diversity, probiotics, type 1 diabetes, type 2 diabetes

## Abstract

Diabetes mellitus represents a significant global health challenge, characterized by impaired insulin production and action, leading to elevated blood glucose levels. This systematic review investigates the association between gut microbiota composition and diversity, along with the structural and functional characteristics of the gut microbiome, and their implications for the risk, prevention, and management of both type 1 diabetes mellitus (T1DM) and type 2 diabetes mellitus (T2DM). Following the Preferred Reporting Items for Systematic Reviews and Meta-Analyses (PRISMA) 2020 guidelines, a comprehensive search across multiple databases yielded 16 studies that met the inclusion criteria. The findings highlight the potential of gut microbiota interventions, such as fecal microbiota transplantation and probiotic supplementation, in improving metabolic parameters and glycemic control. Notably, the review underscores the importance of dietary interventions and the role of specific microbial populations in influencing diabetes outcomes. Despite the promising results, the variability in study designs, sample sizes, and methodologies poses challenges for generalizability and interpretation. This review emphasizes the need for further research to elucidate the mechanisms underlying these associations and to explore personalized microbiome-based therapies in diabetes management. The insights gained could pave the way for innovative therapeutic strategies aimed at harnessing gut health to mitigate the burden of diabetes mellitus.

## Introduction and background

Diabetes mellitus is a chronic disease that occurs when the pancreas does not produce enough insulin or when the body cannot effectively use the insulin it produces, leading to elevated blood glucose levels. This condition encompasses two primary types: type 1 diabetes mellitus (T1DM), characterized by deficient insulin production due to autoimmune destruction of pancreatic cells, and type 2 diabetes mellitus (T2DM), which involves insulin resistance and insufficient insulin production [1]. Together, these types affect over 422 million people globally, with prevalence rising more rapidly in low- and middle-income countries compared to high-income nations. In 2019, diabetes was directly responsible for approximately 1.5 million deaths, with an additional 460,000 deaths attributed to kidney disease resulting from diabetes [1]. Furthermore, diabetes is a major cause of serious health complications, including blindness, kidney failure, heart attacks, and strokes. The global mortality rate from diabetes increased by 3% from 2000 to 2019, highlighting the urgent need for effective prevention strategies [2].

The pathogenesis of diabetes is complex and involves a multitude of factors, including genetics, infections, immunity, obesity, and diet. Current management strategies primarily focus on controlling symptoms, not preventing the disease or its complications. Recently, the gut microbiome has emerged as a potential player in diabetes development [3]. High-throughput sequencing has revealed a diverse gut microbiota with over 1,000 bacterial species. These microbes influence digestion, immunity, and even the production of beneficial compounds. Disruption of this ecosystem (dysbiosis) is linked to various health problems, including diabetes [3]. Elucidating the gut microbiome's role in diabetes may lead to novel therapeutic approaches.

Recent studies have increasingly highlighted the relationship between gut microbiota and diabetes, particularly T1DM and T2DM. Studies suggest that specific bacterial populations may influence diabetes risk or progression [4,5]. Fecal transplants even show promise in altering gut microbiota and potentially preserving insulin production in newly diagnosed diabetics [6]. Understanding this link between gut health and diabetes may lead to novel diagnostic tools and therapeutic strategies.

Our systematic review aims to synthesize these recent findings and further explore the intricate relationship between gut microbiota and diabetes, focusing on potential therapeutic implications and the mechanisms underlying these associations.

## Review

Methods

Following the Preferred Reporting Items for Systematic Reviews and Meta-Analyses (PRISMA) 2020 guidelines, this systematic review investigated the relationship between gut microbiota and diabetes mellitus using the patient, intervention, comparison, outcome, and time (PICOT) framework to define the research question [7]. The target population encompassed individuals diagnosed with either T1DM or T2DM, with no restrictions on age or demographics. The intervention of interest involved evaluating the gut microbiota's composition, diversity, structure, and function. A comparison group of healthy controls, free from diabetes, was used. The review aimed to identify relevant studies that explored the risk of developing diabetes, prevention and management strategies, and the impact of gut microbiota on glycemic control, insulin sensitivity, and other metabolic parameters. No specific timeframe limitations were applied regarding study publication dates.

To ensure a high-quality and focused review, a set of eligibility criteria was established. These criteria ensured a comprehensive, yet focused analysis of the current research on gut microbiota and its potential role in diabetes mellitus, as outlined in Table 1.

**Table 1:** Eligibility criteria T1DM: Type 1 diabetes mellitus; T2DM: Type 2 diabetes mellitus; RCTs: Randomized controlled trials

Inclusion criteria	Exclusion criteria
Human participants	Non-human participants (animal models)
Studies on T1DM and T2DM (particularly risk, prevention, and management)	Studies with unclear or mixed diagnoses of diabetes type
Time frame: no restrictions on the publication date	-
Study design: both RCTs and observational studies (case-control and cohort studies)	Study design: studies did not meet the specified design criteria
Publication type: peer-reviewed articles from academic journals and grey literature reviews of high-quality	Publication type: reviews, systematic reviews, case reports, abstracts, editorials, commentaries, and unfinished trials
Language: English articles with full-text availability for efficient review and analysis	Language: Non-English articles or those without available English translations

Search Process

The literature search was conducted from July 4, 2024, to July 11, 2024, utilizing multiple databases, including PubMed/Medline, Cochrane Central Register of Controlled Trials (CENTRAL), ScienceDirect, Europe PubMed Central (PMC), ClinicalTrials.gov, and Elton B. Stephens Company (EBSCO) Open Dissertations. The search terms were carefully selected based on synonyms for gut microbiota, gut microbiome, T1DM, and T2DM, as outlined in the search strategy in Table 2.

**Table 2:** Search strategy CENTRAL: Cochrane Central Register of Controlled Trials (CENTRAL); European PMC: European PubMed Central; MeSH: Medical Subject Headings; RCT: Randomized Control Trial; EBSCO Open Dissertation: Elton B. Stephens Company Open Dissertation

Search strategy	Databases/registers	No. of studies before/after filters	Filters applied
Date of Search: 04/07/2024; Intestinal microbiota[Title/Abstract] OR Gastrointestinal microbiota[Title/Abstract] OR Gut flora[Title/Abstract] OR Intestinal flora[Title/Abstract] OR Gut microbial community[Title/Abstract] OR Gut bacterial community[Title/Abstract] OR ( "Gastrointestinal Microbiome/genetics"[Mesh] OR "Gastrointestinal Microbiome/physiology"[Mesh] ) OR Intestinal microbiome[Title/Abstract] OR Gastrointestinal microbiome[Title/Abstract] OR Gut microbial genome[Title/Abstract] OR Intestinal microbial genome[Title/Abstract] OR Gut metagenome[Title/Abstract] OR Intestinal metagenome[Title/Abstract] OR (( "Gastrointestinal Microbiome/genetics"[Mesh] OR "Gastrointestinal Microbiome/physiology"[Mesh] )) AND "Gastrointestinal Microbiome"[Mesh] AND Diabetes Mellitus, Type 1 DMT1[Title/Abstract] OR Type 1 Diabetes Mellitus T1DM[Title/Abstract] OR Juvenile Onset Diabetes[Title/Abstract] OR Immune Mediated Diabetes[Title/Abstract] OR Insulin Dependent Diabetes Mellitus[Title/Abstract] OR Juvenile Diabetes[Title/Abstract] OR Insulin Dependent Diabetes[Title/Abstract] OR Ketosis Prone Diabetes[Title/Abstract] OR "Diabetes Mellitus, Type 1"[Mesh] OR Diabetes Mellitus, Type 2 DMT2 OR Type 2 Diabetes Mellitus T2DM OR Adult Onset Diabetes Mellitus OR Non Insulin Dependent Diabetes Mellitus OR NIDDM OR ("Diabetes Mellitus, Type 1"[Mesh]) AND ( "Diabetes Mellitus, Type 2/complications"[Mesh] OR "Diabetes Mellitus, Type 2/enzymology"[Mesh] OR "Diabetes Mellitus, Type 2/metabolism"[Mesh] OR "Diabetes Mellitus, Type 2/therapy"[Mesh] )	PubMed/Medline	701/42	Full text, clinical study, clinical trial, equivalence trial, observational study, randomized controlled trial, humans, English
Date Run: 04/07/2024 13:26:23; Comment: ID Search Hits #1 ''Intestinal NEAR microbiota'' OR ''Gastrointestinal NEXT microbiota'' OR ''Gut flora'' OR ''Intestinal NEXT flora'' OR ''Gut microbial community'' OR ''Gut NEXT bacterial NEXT community'' 5191 #2 ''Intestinal NEAR microbiome'' OR ''Gastrointestinal NEXT microbiome'' OR ''Gut NAER microbial NEAR genome'' OR ''Intestinal NAER microbial genome'' OR ''Gut NEAR metagenome'' OR ''Intestinal near metagenome'' 2174 #3 MeSH descriptor: [Gastrointestinal Microbiome] explode all trees 1721 #4 #2 OR #3 2174 #5 #1 OR #4 6203 #6 ''Diabetes Mellitus, Type 1'' OR ''DMT1'' OR ''Type 1 NEXT Diabetes Mellitus'' OR ''Juvenile Onset NEXT Diabetes'' OR ''Immune Mediated Diabetes'' OR ''Insulin Dependent Diabetes Mellitus'' OR ''Juvenile Diabetes'' OR ''Insulin Dependent Diabetes'' OR ''Ketosis Prone Diabetes'' 73345 #7 MeSH descriptor: [Diabetes Mellitus, Type 1] explode all trees 7612 #8 #6 OR #7 73346 #9 ''Diabetes Mellitus, Type 2'' OR ''DMT2'' OR ''Type 2 NEXT Diabetes Mellitus'' OR ''Adult Onset NEXT Diabetes Mellitus'' OR ''Non Insulin Dependent NEXT Diabetes Mellitus'' OR ''NIDDM'' 81134 #10 MeSH descriptor: [Diabetes Mellitus, Type 2] explode all trees 26412 #11 #9 OR #10 81135 #12 #5 AND #11 432	Cochrane Library (CENTRAL)	432/413	English, trials
Date of search - 07/07/2024 (("intestinal microbiota" OR "gastrointestinal microbiota" OR "gut flora" OR "intestinal flora" OR "gut microbial community" OR "gut bacterial community") OR ("intestinal microbiome" OR "gastrointestinal microbiome" OR "gut microbial genome" OR "intestinal microbial genome" OR "gut metagenome" OR "intestinal metagenome")) AND (("Diabetes Mellitus, Type 1" OR "Type 1 Diabetes Mellitus" OR "Juvenile Onset Diabetes" OR "Immune Mediated Diabetes" OR "Insulin Dependent Diabetes Mellitus" OR "Juvenile Diabetes" OR "Insulin Dependent Diabetes" OR "Ketosis Prone Diabetes") OR ("Diabetes Mellitus, Type 2" OR "Type 2 Diabetes Mellitus" OR "Adult Onset Diabetes Mellitus" OR "Non Insulin Dependent Diabetes Mellitus" OR "NIDDM")) AND ("original study" OR "observational study" OR "interventional study" OR "clinical trial") NOT "review" NOT "meta-analysis"	Europe PMC	169/150	Free full text
Date of search - 11/07/2024 (Gut Microbiota) AND (Diabetes Mellitus, Type-1) OR (Diabetes Mellitus, Type-2) AND free full text NOT (''review'' OR ''Meta analysis'') NOT non-human participants	ScienceDirect	2165/225	Research articles, fields: medicine and dentistry, immunology and microbiology. Language: English open access and open archive
Date of search - 05/07/2024; Condition - Diabetes Mellitus, Type 1 DMT1 OR Type 1 Diabetes Mellitus T1DM OR Juvenile Onset Diabetes OR Immune Mediated Diabetes OR Insulin Dependent Diabetes Mellitus OR Juvenile Diabetes OR Insulin Dependent Diabetes OR Ketosis Prone Diabetes AND Diabetes Mellitus Type 2 DMT2 OR Type 2 Diabetes Mellitus T2DM OR Adult Onset Diabetes Mellitus OR Non Insulin Dependent Diabetes Mellitus OR NIDDM intervention/treatment- Intestinal microbiota OR Gastrointestinal microbiota OR Gut flora OR Intestinal flora OR Gut microbial community OR Gut bacterial community OR Intestinal microbiome OR Gastrointestinal microbiome OR Gut microbial genome OR Intestinal microbial genome OR Gut metagenome OR Intestinal metagenome	ClinicalTrials.gov register	92/2	Completed with results
Date of Search - 11/07/2024 ((Intestinal microbiota OR Gastrointestinal microbiota OR Gut flora OR Intestinal flora OR Gut microbial community OR Gut bacterial community) OR (Intestinal microbiome OR Gastrointestinal microbiome OR Gut microbial genome OR Intestinal microbial genome OR Gut metagenome OR Intestinal metagenome)) AND (Diabetes Mellitus, Type 1 DMT1 OR Type 1 Diabetes Mellitus T1DM OR Juvenile Onset Diabetes OR Immune Mediated Diabetes OR Insulin Dependent Diabetes Mellitus OR Juvenile Diabetes OR Insulin Dependent Diabetes OR Ketosis Prone Diabetes) OR (Diabetes Mellitus, Type 2 DMT2 OR Type 2 Diabetes Mellitus T2DM OR Adult Onset Diabetes Mellitus OR Non Insulin Dependent Diabetes Mellitus OR NIDDM) AND (RCT OR trial OR Cohort OR observational study OR clinical study OR Case control) NOT (review OR Meta analysis)	EBSCO Open Dissertation	907/113	Dissertations, (RCT OR trial OR Cohort OR observational study OR Clinical study OR Case control) NOT review OR meta analysis

Screening and Quality Assessment

A comprehensive two-stage screening process was implemented, utilizing the Rayyan app® (Qatar Computing Research Institute, Qatar) for effective record management [8]. In the initial stage, a single reviewer evaluated titles and abstracts to identify potentially relevant studies. In the subsequent stage, two independent reviewers examined the full-text articles against the established inclusion and exclusion criteria. Any disagreements between the reviewers were resolved either through consensus or by involving a third reviewer for arbitration.

To assess the quality of the studies, the Newcastle-Ottawa Scale (NOS) [9] was utilized for observational studies, the ROBINS-I tool for non-randomized clinical trials (RCTs) [10], and the Cochrane Risk-of-Bias tool (RoB 2) for RCTs [11]. Two independent reviewers conducted the quality assessments, with discrepancies addressed through consensus or arbitration. Studies were classified as having good, fair, or poor quality based on the overall evaluation of the RoB.

Data Synthesis

The extracted data were synthesized according to the design and outcome measures of the included studies. Given the expected heterogeneity in study designs and methodologies, a narrative synthesis approach was employed.

Results

**Figure 1:**
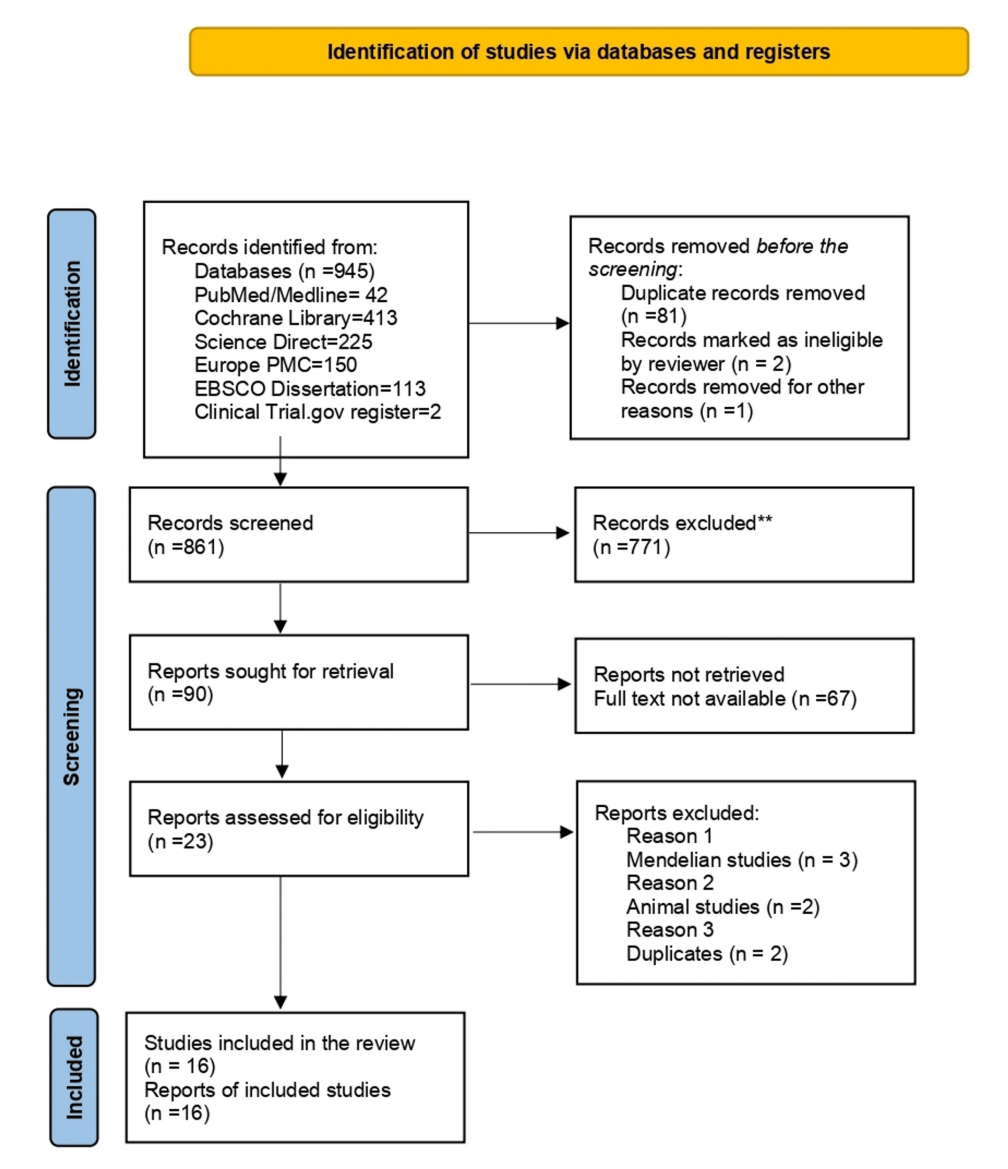
PRISMA diagram PRISMA: Preferred Reporting Items for Systematic Reviews and Meta-Analyses

A comprehensive search strategy, detailed in the Methods section, yielded 945 initial records from multiple databases and registers. After removing duplicates and applying eligibility criteria, 23 full-text articles were retrieved for detailed assessment. Ultimately, 16 studies met the inclusion criteria and were included in the final review. The PRISMA flow diagram (Figure 1) demonstrates the screening process done to include the 16 studies for future review. 

RoB Assessment

Out of the 12 RCTs assessed using the Cochrane RoB 2 tool [11], specifically the studies by de Groot et al. (2021), Wu et al. (2022), Zhang et al. (2020), Palacios et al. (2020), Wang et al. (2022), Razmpoosh et al. (2019), and Mobini et al. (2017), Burton et al. (2015) demonstrated consistent strengths across key quality domains, with each study meeting high standards for criteria such as randomization, blinding, and outcome reporting [6,11-18].

Four studies exhibited some concerns regarding bias in various domains. Deng et al. (2022) presented some concerns due to its open-label design, which could affect patient compliance and perceptions of treatment effectiveness, although no differential adherence was reported [19]. Specifically, Zhao et al. (2018) raised concerns about domain 2 due to its open-label design, which could potentially influence participants' adherence and reporting. However, the standardization of treatment variability was maintained, as both groups received acarbose [20]. Su et al. (2022) highlighted concerns regarding deviations from intended interventions, as participants did not strictly adhere to dietary protocols, and a higher dropout rate was observed in the dietary fiber group [21].

Lastly, Shabani-Mirzaee et al. (2023) indicated concerns related to deviations from intended interventions, as the probiotic group received both probiotics and insulin, while the control group received insulin alone. Although single blinding was implemented for participants, the lack of blinding for those administering the interventions posed a RoB [22]. These findings underscore the necessity of addressing potential biases to enhance the validity of RCT outcomes.

Table 3 below presents the details of the quality assessment conducted for the RCTs. This table outlines the specific biases identified in each study, providing a comprehensive overview of the assessment results.

**Table 3:** Risk of Bias Assessment of Randomized Clinical Trials: Cochrane Risk-of-Bias Tool (RoB 2) Note: RoB 2 domains: 1) Randomization process, 2) Deviations from intended interventions, 3) Missing outcome data, 4) Measurement of outcome, 5) Selection of reported result +, Low risk of bias; -, High risk of bias; !, Some concerns about risk of bias

Study by/year of publication	Domain 1	Domain 2	Domain 3	Domain 4	Domain 5	Overall
de Groot et al. (2021) [6]	+	+	+	+	+	+
Wu et al. (2023) [12]	+	+	+	+	+	+
Zhang et al. (2020) [13]	+	+	+	+	+	+
Palacios et al. (2020) [14]	+	+	+	+	+	+
Wang et al. (2022) [15]	+	+	+	+	+	+
Razmpoosh et al. (2019) [16]	+	+	+	+	+	+
Mobini et al. (2017) [17]	+	+	+	+	+	+
Burton et al. (2015) [18]	+	+	+	+	+	+
Deng et al. (2022) [19]	+	!	!	+	+	!
Zhao et al. (2018) [20]	+	!	+	+	+	!
Su et al. (2022) [21]	+	!	!	+	+	+
Shabani-Mirzaee et al. (2023) [22]	+	!	+	+	+	!

The quality assessment of four non-RCTs, using the ROBINS-I tool, revealed notable concerns regarding potential biases [10].

In Abo Ali et al. (2013), bias due to confounding was a concern due to a small sample size and the absence of a control group, which limited the ability to address confounding factors effectively. Additionally, selection bias was evident, as only 14 out of the eligible participants completed the study [23]. Paun et al. (2019) also exhibited concerns related to confounding, as the study compared T1DM patients and healthy controls, accounting for human leukocyte antigen (HLA) haplotypes but not other potential confounders. The selection of participants from specific pediatric cohorts raised further questions about representativeness [24]. Bryrup et al. (2019) highlighted concerns in the classification of interventions and outcome reporting due to the non-blinded design, which could bias results. Additionally, the study noted that some participants had to reduce their metformin intake, potentially confounding outcomes while missing data from dropouts was not adequately addressed [25].

Lastly, Lee et al. (2021) raised concerns regarding outcome measurement, as established methods were used; however, the subjective nature of self-reported adherence could introduce bias. The small sample size of 20 participants further exacerbated potential selection bias [26]. Overall, these findings emphasize the need to address biases in non-RCTs to ensure the validity of their results. Details are presented in Table 4.

**Table 4:** Risk of Bias Assessment: Risk of Bias in Non-randomized Studies - of Interventions (ROBINS-I) Tool Note: ROBINS-I domains - 1) Bias arising from confounding, 2) Bias arising from selection of participants, 3) Bias arising from classification of interventions, 4) Bias arising from deviations from intended interventions, 5) Bias arising from missing data, 6) Bias in measurement of outcomes, 7) Bias in selection of reported results +, Low risk of bias; -, High risk of bias; !, Some concerns about risk of bias

Study/year	Domain 1	Domain 2	Domain 3	Domain 4	Domain 5	Domain 6	Domain 7	Overall
Abo Ali et al. (2013) [23]	!	!	+	+	+	+	!	!
Paun et al. (2019) [24]	!	!	+	+	+	+	+	!
Bryrup et al. (2019) [25]	+	+	!	!	!	+	+	!
Lee et al. (2021) [26]	+	!	+	+	+	!	+	!

Characteristics of Included Studies

Out of the studies reviewed, a total of 16 studies were identified. Among these, 12 were RCTs, while four were observational studies or clinical trials. The majority of the studies were conducted in China, with a notable concentration of research efforts in this region. Other studies were distributed across various countries, including the Netherlands, Australia, Iran, Sweden, Egypt, Canada, Denmark, and South Korea. This geographical distribution highlights a diverse range of research environments and practices in the investigation of diabetes management and gut microbiota interventions. Clear details regarding the included studies are provided in Table 5.

**Table 5:** Summary of included studies FMT: Fecal microbiota transplantation; FBG: Fasting blood glucose; PBG: Post-prandial blood glucose; HbA1c: Hemoglobin A1c; HOMA-IR: Homeostasis model assessment for insulin resistance; BMI: Body mass index; HOMA-BCI: Homeostasis model assessment of beta cell function; AUC: Area under curve; BBR: Berberine; PROB+BBR: Probiotic+berberine; TGs: Triglycerides; TC: Total cholesterol; LDL cholesterol: Low density lipo-protein cholesterol; T2DM: Type-2 diabetes mellitus; SCFA: Short chain fatty acid; T1DM: Type-1 diabetes mellitus; HDL: High density lipo-protein; SBP: Systolic blood pressure; DBP: Diastolic blood pressure; NGS: Next generation sequencing; IgG1 ACab: Immunoglobulin G1 anti-commensal antibody; IgG2 ACab: Immunoglobulin G2 anti-commensal antibody; ICA: Islet cell autoantibody; IA2A: Insulinoma-2-associated autoantibody; PCoA: Principal coordinates analysis; GIMM: Gastrointestinal microbiome modulator

Author/year	Study design	Country	Aim of study	Sample size	Mean age	Key findings
Wu et al. (2023) [12]	RCT	China	To evaluate adjunctive FMT with metformin in the southeast Chinese population with type-2 DM	36	n/a	Shows significant improvements in FBG, PBG, HbA1c, and HOMA-HBCI (p < 0.05). Both the FMT-only and FMT plus metformin groups had significant reductions in HOMA-IR and BMI (p < 0.05), whereas the metformin-only group did not show significant changes after 4 weeks.
de Groot et al. (2021) [6]	RCT	Netherlands	To study the microbiota-mediated effects on disease progression in type-1 DM using FMT	34	Autologous group - 25(21.5-28.5); Allogeneic group - 24.3(18.9-29.7)	Change in fasting C-peptide over time p value = 0.00019; Change in C-peptide AUC over time p-value = 0.000067; Change in HbA1c over time over time p-value = 0.12
Zhang et al. (2020) [13]	RCT	China	To find an effective strategy for treating type-2 DM patients using probiotics and berberine by altering gut microbiome dysbiosis and comparing their efficacy	566	Placebo: 54(46-61); probiotics: 54(46-59); berberine: 53(42-61); probiotic + berberine: 53.5(47-60)	BBR-containing treatments improved metabolic parameters like FBG, PBG, TGs, TC, and LDL cholesterol. Prob + BBR significantly reduced HOMA-IR, but BBR alone did not. Both BBR groups reported more gastrointestinal side effects, while glycemic control stayed stable.
Palacios et al. (2020) [14]	RCT pilot study	Sydney, Australia	To assess the effectiveness of an evidence-based multi-strain probiotic on glycemia, intestinal permeability, and inflammatory markers in adults with pre-diabetes and early T2DM, and to determine if the probiotic had an additional impact on glycemia in patients on metformin	80	Probiotic: 61.4(52.5-70.3); placebo: 56.1(43.8-68.4)	No significant changes in metabolic, inflammatory, and permeability markers were observed between the probiotic and placebo groups. Patients on probiotic + metformin showed significant improvements (p < 0.05) in fasting plasma glucose, insulin resistance, and the permeability marker zonulin.
Zhao et al. (2018) [20]	RCT	China	To assess whether a high-fiber diet alters the gut microbiota and enhances glucose homeostasis in individuals with T2DM.	33	n/a	The acetate SCFA producer index was negatively correlated with HbA1c at baseline and after the intervention, emphasizing the role of SCFA producers in managing T2DM with fermentable carbohydrates. A deficiency in beneficial gut functions, like SCFA production from carbohydrate fermentation, may contribute to chronic diseases such as T2DM. This SCFA production is an essential "ecosystem service" provided by gut microbiota to support human health.
Razmpoosh et al. (2019) [16]	RCT	Tehran	Investigating the effect of multi-strain probiotics on fasting plasma glucose, plasma insulin, and lipid profile	68	58.6(52.1-65.1)	Fasting plasma glucose significantly decreased (p = 0.01), while HDL cholesterol significantly increased (p = 0.002). No significant changes were observed in insulin, triglycerides, total cholesterol, insulin resistance, or anthropometric measurements (weight, waist circumference, and BMI).
Mobini et al. (2017) [17]	RCT	Gothenburg, Sweden	To investigate the metabolic effects of 12-week oral supplementation with *Lactobacillus reuteri* DSM 17398 with T2DM patients on insulin therapy	46	*L. reuteri* low = 66(60-72); *L. reuteri *high = 64(58-70)	Probiotic intervention led to a significant reduction in HbA1c levels. No significant changes were observed in weight, body composition, blood pressure, lipid profile, liver function, or adipokines. While total bile acid levels remained unchanged, unconjugated bile acids increased, with a notable rise in secondary deoxycholic acid in the high-dose group.
Abo Ali et al. (2013) [23]	Clinical trial	Egypt	To assess the impact of intestinal microflora on the phagocytic activity of polymorphonuclear leukocytes in patients with diabetes	40	52.7	In uncontrolled diabetes, *Lactobacillus acidophilus* counts were significantly higher compared to controlled diabetes. There was a notable positive correlation between *L. acidophilus* counts and HbA1c, FBG, and PBG in uncontrolled diabetics. Chronic hyperglycemia can impair a wide range of neutrophil functions including chemotaxis, adherence, phagocytosis, and intracellular killing. Highly significant negative correlation between phagocytic index with HbA1c and a significant negative correlation with the FBG and PPBG in uncontrolled diabetics.
Shabani-Mirzaee et al. (2023) [22]	RCT	Bahrami children's hospital, Tehran	To evaluate the effect of oral consumption of probiotics on glycosylated hemoglobin in children with T1DM	52	9.3(6.4-12.2)	The mean FBG was significantly lower in the probiotic group than the control group (p = 0.016). The decrease in HbA1C was not clinically significant (p = 0.692). No significant changes in total cholesterol (p = 0.920), LDL (p = 0.597), HDL (p = 0.684), or triglyceride levels (p = 0.500).
Deng et al. (2022) [19]	RCT	Henan, China	To assess if the cardiovascular benefits of empagliflozin are linked to changes in gut bacteria and plasma metabolites, and to explore its potential as an initial treatment for T2DM patients at risk of cardiovascular diseases	76	18-70 years	Significant reductions were observed in HbA1c (p < 0.0001); PBG (p < 0.0001); body weight (p < 0.0001); SBP (p = 0.0002); DBP (p = 0.0053); uric acid (p = 0.0005); increases in hematocrit (p < 0.0001) and adipokine (p = 0.0001) were noted only in patients on empagliflozin.
Wang et al. (2022) [15]	RCT	China	To enhance glycemic control in T1DM patients by reducing inflammatory cytokines through daily use of probiotic strains, specifically *Lactobacillus salivarius* and *Bifidobacterium animalis*	56	Probiotic = 14.1; placebo = 14.3	NGS revealed changes in gut bacteria before and after the intervention, with *Firmicutes* being the most prevalent in the probiotic group, followed by *Actinobacteria*. The average FBG and HbA1c significantly decreased in the probiotic group compared to the placebo group (p = 0.000). Three months post-intervention, FBG and HbA1c remained lower in the probiotic group (p = 0.0001 and p = 0.038, respectively).
Su et al. (2022) [21]	RCT	China	To evaluate the safety and efficacy of a new diet containing probiotics, prebiotics, and whole grains (PPW formulation) and FMT combination treatment method of T2DM	25	Diet only (D) = 60.4; Diet + FMT (DF) = 57	Both groups significantly reduced BMI (p < 0.05), with the FMT group showing faster weight loss. FMT also led to more pronounced decreases in blood sugar (FBG and HbA1c) and blood pressure (SBP) compared to the control group.
Paun et al. (2019) [24]	Clinical trial	Canada	To evaluate whether there is any association of HLA-dependent islet autoimmunity with systemic antibody responses to intestinal commensal bacteria in children with T1DM	Cases = 68; controls = 62	10.68 years	The isotype of anti-commensal antibody (ACab) responses clustered with the islet cell autoantibody (ICA) specificity. Specifically, IgG1 ACab responses were negatively correlated with ICA, and IgG2 ACab responses were positively correlated with IA2A and negatively correlated with anti-insulin autoantibodies in seroconverted individuals.
Bryrup et al. (2019) [25]	Clinical trial	Denmark	1) To study metformin-induced compositional changes in non-diabetic men; 2) To investigate whether the gut microbiota composition before treatment was associated with the gastrointestinal side effects of metformin therapy	27	26 years (18-35)	Metformin treatment temporarily altered gut microbiota composition. Eleven bacterial genera experienced fluctuations, with *Intestinibacter* and *Clostridium* decreasing and *Escherichia/Shigella* and *Bilophila* increasing. Changes were reversible upon treatment cessation. Metformin's impact on the gut microbiome was independent of diabetic status.
Lee et al. (2021) [26]	Clinical trial	Korea	To assess the hypoglycemic effects of metformin and to explore the underlying mechanisms of these effects using metagenomic and global metabolomic approaches	20	19-33 years	After metformin administration, bacterial genera abundances changed, with *Escherichia* potentially contributing to its hypoglycemic effect. Alpha diversity significantly increased (p = 0.043), and PCoA analysis showed higher beta diversity post-treatment (p < 0.004).
Burton et al. (2015) [18]	RCT	Louisiana, United States	To evaluate that the addition of a gastrointestinal microbiome modulator to metformin improves metformin tolerance and fasting glucose levels	11	43.2	The mean fasting glucose was significantly (p < 0.02) lower with the metformin-GIMM combination. Volunteers experienced significantly fewer gastrointestinal side effects from metformin when it was combined with GIMM compared to when it was combined with a placebo.

Discussion

The role of gut microbiota in diabetes mellitus has garnered significant attention due to its potential impact on disease management and therapeutic outcomes. This discussion synthesizes findings from 16 studies that explored various microbiome-based interventions for diabetes, providing insights into age-related trends, study aims, and key findings.

Age-Related Insights

The studies reviewed feature a wide age range, with participants from children to older adults. For instance, Shabani-Mirzaee et al. (2023) focused on children with T1DM, revealing that probiotics significantly reduced fasting blood glucose (FBG) levels, though changes in hemoglobin A1c (HbA1c) were not clinically significant [22]. In contrast, studies involving older adults, such as Palacios et al. (2020), indicated that the mean age of participants was over 60, highlighting the need for tailored interventions in older populations where metabolic responses may differ [14].

Interestingly, younger participants, such as those in the studies by Bryrup et al. (2019) and Lee et al. (2021), showed significant changes in gut microbiota composition due to metformin treatment, emphasizing the drug's impact on younger, non-diabetic individuals [25,26]. This suggests that age may influence how gut microbiota responds to interventions, necessitating further research into age-specific responses to microbiome-based treatments.

Several interesting findings emerged from the reviewed studies, shedding light on the potential of microbiome-based interventions in diabetes management.

Fecal Microbiota Transplantation (FMT) and Metformin Synergy

Wu et al. (2023) demonstrated that adjunctive FMT combined with metformin improved FBG, postprandial blood glucose (PBG), HbA1c, and homeostasis model assessment for insulin resistance (HOMA-IR) more effectively than metformin alone. This underscores the potential of FMT to enhance metabolic control in T2DM [12]. Additionally, de Groot et al. (2021) showed that FMT influenced disease progression in T1DM by modifying fasting C-peptide and C-peptide area under the curve (AUC), though the changes in HbA1c were not statistically significant [6].

Probiotic Efficacy

Zhang et al. (2020), Razmpoosh et al. (2019), and Mobini et al. (2017) observed improvements in secondary metabolic parameters and fasting plasma glucose with probiotic and berberine treatments. However, the combination of probiotics and berberine showed more pronounced effects on insulin resistance compared to berberine alone [13,16,17]. Palacios et al. (2020) found no significant improvements in metabolic and inflammatory markers with probiotic use alone, but notable benefits when combined with metformin [14].

Dietary Interventions

Dietary interventions also emerged as a crucial factor in managing diabetes. Zhao et al. (2018) emphasized the importance of high-fiber diets in promoting the production of short-chain fatty acids (SCFAs), which play a vital role in glucose homeostasis [20]. This finding aligns with the increasing recognition of dietary strategies as integral components of diabetes management, suggesting that lifestyle modifications can significantly impact metabolic health [20].

Specific Probiotic Strains and Long-Term Effects

Mobini et al. (2017) reported a significant reduction in HbA1c with *Lactobacillus reuteri*, though no effects on weight or other metabolic parameters were noted [17]. Wang et al. (2022) demonstrated that specific probiotic strains led to significant reductions in FBG and HbA1c, suggesting a beneficial role in long-term glycemic control [15].

FMT and Diet Combinations

Su et al. (2022) showed that combining a diet with probiotics, prebiotics, and FMT resulted in more significant weight loss and improvements in FBG and HbA1c compared to diet alone, highlighting the synergistic effects of dietary and microbiome interventions [21].

Metformin and Gut Microbiota Changes

Lee et al. (2021) and Bryrup et al. (2019) observed that metformin alters gut microbiota composition and diversity, potentially contributing to its hypoglycemic effects [26,25]. Notably, Lee et al. (2021) linked changes in bacterial genera to improved glycemic control via metabolic pathways [26].

Impact on Immune Function

Another significant insight came from Abo Ali et al. (2013), who revealed a noteworthy correlation between *Lactobacillus acidophilus* counts and metabolic parameters in individuals with uncontrolled diabetes. This suggests that gut microbiota may influence immune function and contribute to hyperglycemia, highlighting the interconnectedness of gut health and metabolic regulation [23].

Adverse Effects Mitigation

Burton et al. (2015) showed that combining metformin with a gastrointestinal microbiome modulator (GIMM) improved metformin tolerance and reduced gastrointestinal side effects, underscoring the importance of managing treatment-related adverse effects [18].

Association of Gut Microbiota and Autoimmunity in T1DM

Paun et al. (2019) explored the link between HLA-dependent islet autoimmunity and antibody responses to gut bacteria in children with T1DM [24]. Involving 68 cases and 62 controls, the study found that anti-commensal antibody (ACab) responses correlated with islet cell autoantibody (ICA) specificity. Specifically, IgG1 ACab responses were negatively correlated with ICA, while IgG2 ACab responses showed positive correlations with insulinoma-associated autoantibody (IA2A) and negative correlations with anti-insulin autoantibodies. These findings suggest that gut microbiota may influence autoimmune responses in T1DM, emphasizing the complex interplay between the microbiome and immune function [24].

Probiotics and Glycemic Control in T1DM

Shabani-Mirzaee et al. (2023) evaluated the effects of probiotics on HbA1c in an RCT study with 52 children (mean age 9.3 years) at Bahrami Children's Hospital, Tehran. While FBG significantly decreased in the probiotic group, the change in HbA1c was not clinically significant. No notable differences were found in cholesterol or triglyceride levels, indicating that while probiotics may lower FBG, their impact on glycemic control remains limited [22].

Cardiovascular Benefits and Gut Microbiota in T2DM

Deng et al. (2022) assessed empagliflozin's cardiovascular benefits in an RCT with 76 participants in Henan, China. The study reported significant reductions in HbA1c, PBG, body weight, systolic and diastolic blood pressure, and uric acid. Additionally, increases in hematocrit and adipokine levels were observed only in those receiving empagliflozin, suggesting a potential link between cardiovascular benefits and gut microbiota modulation in T2DM management [19].

The findings from this systematic review underscore the significant role of gut microbiota diversity in the management and prevention of metabolic disorders, particularly diabetes mellitus. The evidence suggests that interventions targeting gut microbiota, such as FMT and probiotic supplementation, can lead to improvements in various metabolic parameters. However, the variability in study designs and outcomes highlights the need for further research to elucidate the mechanisms underlying these associations.

Clinical implications

The insights gained from this review have important clinical implications. Healthcare providers should consider the gut microbiota as a potential therapeutic target in the management of diabetes mellitus. Personalized interventions that incorporate dietary modifications and microbiome-based therapies may enhance glycemic control and improve patient outcomes. Moreover, understanding the patient's gut microbiome composition could inform treatment strategies and help mitigate the risk of complications associated with diabetes.

Future directions for research

Future research should focus on longitudinal studies that explore the causal relationships between gut microbiota diversity and metabolic disorders. Investigating the specific microbial species and their functional roles in glucose metabolism will be crucial for developing targeted therapies. Additionally, studies should aim to assess the effectiveness of microbiome interventions across diverse populations, taking into account age, sex, and genetic predispositions. Finally, exploring the integration of gut microbiota assessments into routine clinical practice could pave the way for more personalized and effective diabetes management strategies.

Strengths and limitations of the systematic review

Strengths

One of the primary strengths of this systematic review is its comprehensive search strategy, which utilized multiple reputable databases, including PubMed/Medline, CENTRAL, ScienceDirect, Europe PMC, ClinicalTrials.gov, and EBSCO Open Dissertations. This extensive approach ensured a wide coverage of relevant literature, enhancing the review's reliability.

The inclusion of diverse study designs further strengthens the review. By incorporating various types of studies, such as RCTs, controlled clinical trials (CCTs), and observational studies (cohort and case-control), the review provides a more holistic understanding of the gut microbiota's impact on diabetes mellitus. This diversity enriches the findings and allows for a broader perspective on the subject.

A rigorous quality assessment was conducted using established tools, such as the NOS for observational studies, the ROBINS-I tool for non-randomized trials, and the Cochrane RoB tool for RCTs. This thorough evaluation ensures that the included studies met high standards of scientific rigor, contributing to the credibility of the review.

The focused research question also enhances the review's value, as it concentrates on the specific relationship between gut microbiota diversity and diabetes mellitus. This targeted approach provides insights that can inform future research and clinical practice. Furthermore, the review's broad scope, which includes both type 1 and type 2 diabetes, allows for a comprehensive understanding of potential differences in gut microbiota associations across these conditions.

Limitations

Despite its strengths, the review has several limitations. One notable restriction is the focus on studies published in English, which may have excluded relevant research published in other languages. This language restriction could introduce publication bias, limiting the comprehensiveness of the findings.

Additionally, variability in sample sizes among the included studies poses a challenge. Some studies had small sample sizes, which can affect the generalizability of the results. This variability may limit the ability to draw definitive conclusions about the impact of gut microbiota on diabetes.

The heterogeneity in study designs presents another challenge. Differences in methodologies, interventions, and outcome measures across the included studies may hinder direct comparisons and synthesis of results. This heterogeneity complicates the interpretation of findings, making it difficult to establish clear connections.

Concerns regarding bias in the included studies also exist. Several RCTs and observational studies raised issues related to blinding, selection, and confounding factors, which could affect the validity of the reported outcomes. Such biases may undermine the reliability of the conclusions drawn from the review.

Moreover, many studies were short-term interventions, limiting the ability to conclude the long-term effects of gut microbiota on metabolic disorders. This lack of longitudinal data restricts the understanding of how gut microbiota influences diabetes over time.

Finally, while the review primarily highlighted specific interventions, such as probiotics and FMT, it did not extensively explore other potential factors influencing gut microbiota diversity, including diet, lifestyle, and genetics. This oversight may limit the overall understanding of the complex interactions at play.

## Conclusions

The investigation into gut microbiota diversity as a critical factor in the management and prevention of diabetes mellitus reveals significant potential for therapeutic interventions. This systematic review consolidates findings from various studies, demonstrating that alterations in gut microbiota composition can substantially impact metabolic parameters and glycemic control in both type 1 and type 2. Interventions such as FMT and probiotic supplementation show promise in enhancing clinical outcomes, emphasizing the need for microbiome-targeted therapies in diabetes care.

However, the variability in study designs and methodologies presents challenges to the generalizability of these findings. Further research is essential to elucidate the mechanisms linking gut microbiota to diabetes pathophysiology and to explore personalized microbiome-based interventions. Integrating gut microbiota assessments into clinical practice could optimize diabetes management, tailoring strategies to individual microbiome profiles to improve glycemic control and reduce complications associated with the disease.
